# Therapeutic effect of polysaccharide fraction of *Atractylodis macrocephalae Koidz*. in bovine subclinical mastitis

**DOI:** 10.1186/s12917-015-0494-6

**Published:** 2015-07-25

**Authors:** Wei Xu, Ran Guan, Yisong Lu, Xiaoyan Su, Ye Xu, Aifang Du, Songhua Hu

**Affiliations:** Department of Veterinary Medicine, College of Animal Sciences, Zhejiang University, 866 Yu Hang Tang Rd, Hang Zhou, 310058 PR China

**Keywords:** *Atractylodis macrocephalae Koidz*, Polysaccharides, Mastitis, Supramammary lymph node

## Abstract

**Background:**

Mastitis is considered the most significant and persistent disease in dairy cows, bringing about large economic losses. Subclinical mastitis brings about major cost implications, for it is difficult to detect due to absence of any visible indications and can persist in the mammary tissue throughout lactation. Immunomodulators have been widely used to reduce intramammary infections by modulating bovine mammary gland. *Atractylodis macrocephalae Koidz.* polysaccharides (*RAMP*), extracted from herbal medicine, has been used widely especially for its immunomodulatory function for many years. The objective of this study was to estimate an oil emulsified *Atractylodis macrocephalae Koidz.* polysaccharides (*RAMP-O*) as a potential therapeutic agent to treat subclinical mastitis by subcutaneous injection of *RAMP-O* in the area of supramammary lymph node in lactating cows via analysis of SCC, IMIs and NAGase.

**Results:**

Injection of *RAMP-O* in the area of supramammary lymph node significantly reduced milk SCC and NAGase activity compared with control. The quarters with bacterial infection were also progressively reduced in *RAMP-O* treated cows and only 9 quarters were found to have bacterial infection, while no obvious change was found in the control group.

**Conclusions:**

Subcutaneous injection of *RAMP-O* in the area of supramammary lymph node had therapeutic value in the treatment of bovine subclinical mastitis by reducing SCC, NAGase and IMIs in milk. Considering both the therapeutic effect and the cost of *RAMP-O*, 32 mg per dose was found most suitable to reduce milk SCC and NAGase. Therefore, *RAMP-O* deserves further study for its use in treatment of bovine mastitis.

## Background

Mastitis is the most common disease of dairy cows and mainly caused by bacterial infection of the mammary gland [1–3]. Subclinical form cannot be detected by naked eyes with characteristics of increased SCC and decreased milk production [4–7]. In China, 40–80 % of milking cows are subclinically infected, which account for the annual economic loss of more than 900 million dollars [8]. The antibiotic therapy is generally used for the treatment of mastitis, however it may lead to drug resistant bacteria and also attribute to residue in the human food chain [9]. In Sweden, antibiotic treatment during lactation is not recommended in case of subclinical mastitis. Moreover, the use of antibiotic is on prescription only [10]. Therefore, looking for a novel alternative therapy for bovine mastitis is an urgent topic.

Since the end of 1980s, Sordillo and Daley [11] reviewed the use of cytokines as immunomodulators and potential therapeutic agents for mastitis therapy. Recombinant interferon-γ has exhibited effectiveness in experimentally induced *Escherichia coli* and *Staphylococcus aureus* IMIs [12]. The extract from the root of *Panax ginseng* C.A. Meyer has been reported to have immunomodulatory property [13–15]. Intramammary infusion of ginseng saponins (GS) has been found to modulate the immunity of mammary gland by potentiating mRNA expression of proinflammatory cytokines (IL-1α, IL-1β and TNF-α) in cows at drying off [16, 17].

*Atractylodis macrocephalae Koidz.* is a plant of *Compositae* having its natural resource in Zhejiang provinces in China. Its rhizome (*RAM*) has been used as a traditional Chinese medicine for about 2000 years [18]. *RAM* consists of various active fractions, such as polysaccharides, volatile oil and lactones [19]. Previous investigation has shown that oral administration of the extract made from *RAM* has enhanced immune responses of mice. Later investigation has shown that polysaccharides (*RAMP*) extracted from *RAM* is active for the immune enhancement. Recent research has shown that injection of *RAMP* together with foot-and-mouth disease (FMD) vaccine significantly enhanced both humoral and cellular immune responses [20–22]. Therefore, we hypothesized that injection of *RAMP* may be useful in reduction of IMIs by stimulating the immunity in cows. The objective of this study was to estimate an oil emulsified *RAMP* (*RAMP-O*) as a potential therapeutic agent to treat subclinical mastitis by subcutaneous injection of *RAMP-O* in the area of supramammary lymph node in lactating cows via analysis of SCC, IMIs and NAGase.

## Results

### Characterization of *RAMP*

**Fig. 1:**
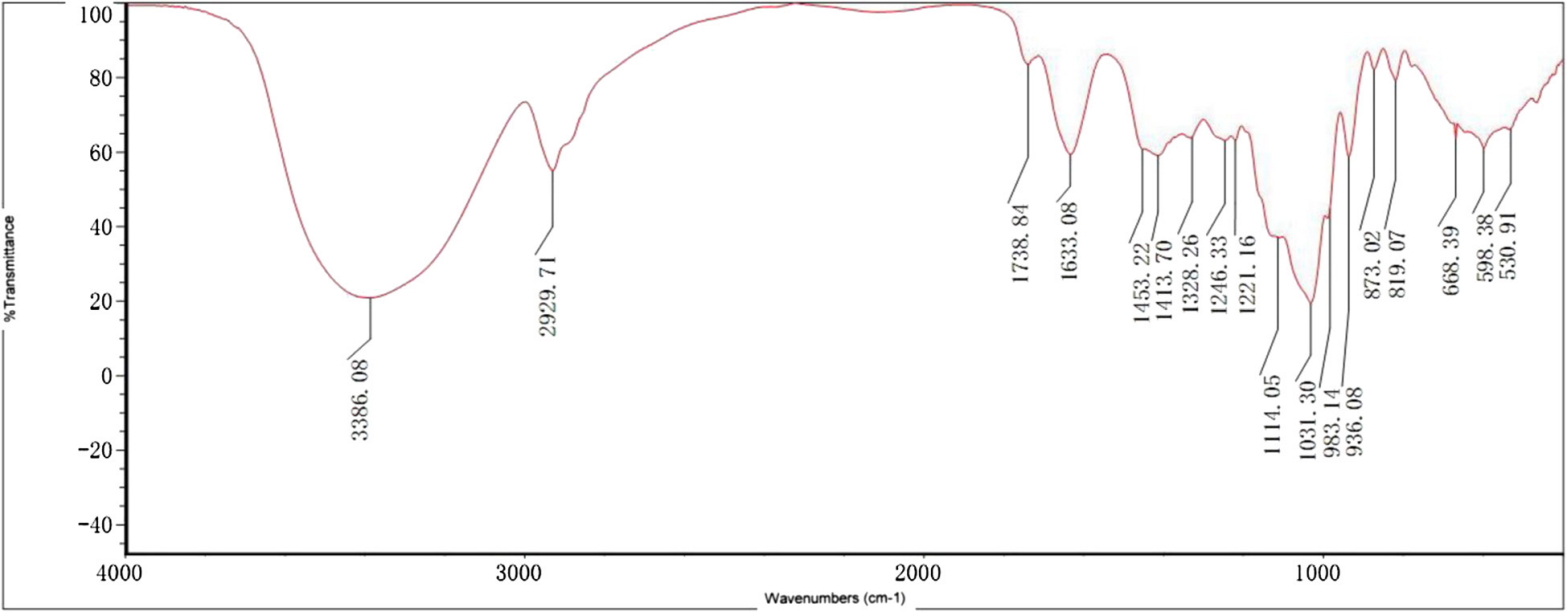
FTIR spectroscopy of *RAMP*. The FITR spectrum of *RAMP* showed a strong broad band of absorption at 3386.08 cm^−1^ attributed to O-H stretching vibration of the polysaccharides. The band at 2929.71 cm^−1^ was ascribed to C-H stretching vibration in carbohydrates and the band at 1633.08 cm^−1^ was due to the presence of bound water. The characteristic peak of absorption at 873.02 cm^−1^ was due to the variable-angle vibration of β-configuration C-H of pyranose. The band at 819.07 cm^−1^ was attributed to the variable-angle vibration of C-H of furan ring. The absorption peak at 350–660 cm^−1^ indicated that it was a pyran-type polysaccharide

**Fig. 2:**
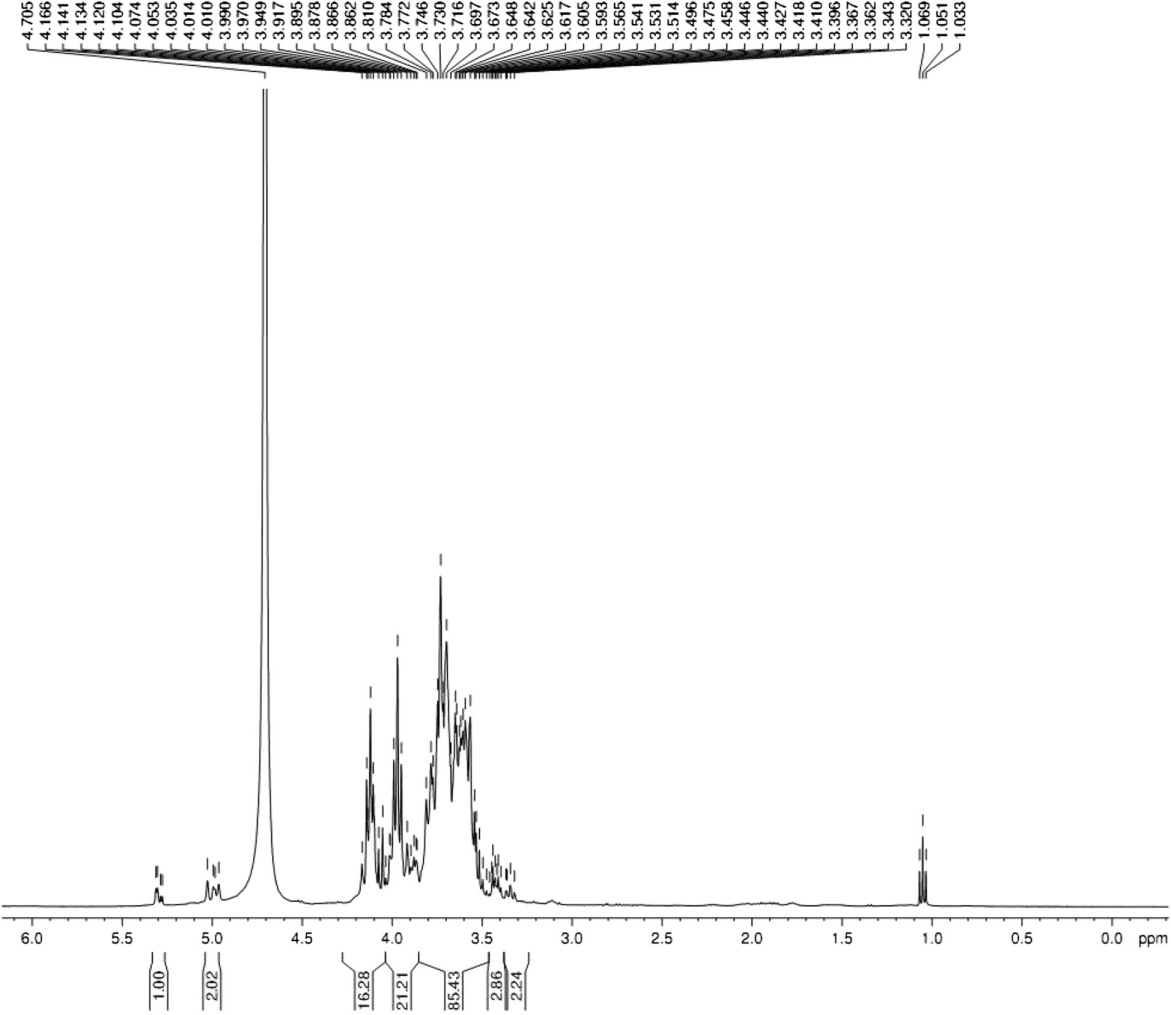
^1^H NMR spectrum of *RAMP.* The ^1^H NMR spectra showed that *RAMP* was a polysaccharide with both α and β configurations, while the β-configuration is dominant

The polysaccharide contained in *RAMP* was 89.63 % as measured by phenol-sulfuric acid method and it did not contain reducing sugar and starch-type polysaccharides on the basis of negative Fehling’s reagent and iodine-potassium iodide reactions. UV analysis and triketohydrindene hydrate reaction showed that *RAMP* was not contaminated with protein. The FTIR spectrum of *RAMP* measured in KBr pellets was shown in Fig. 1. The characteristic strong broad band of absorption at 3386.08 cm^−1^ was attributed to O-H stretching vibration of the polysaccharides. The band at 2929.71 cm^−1^ was ascribed to C-H stretching vibration in carbohydrates and the band at 1633.08 cm^−1^ was due to the presence of bound water. The featured signal ester carbonyl groups at 1738.84 cm^−1^ suggested that *RAMP* was uronic acid-contained polysaccharide. The bands in the range of 1400–1200 cm^−1^ represented the variable-angle vibrations of C-H in polysaccharides. The bands between 1031 and 1150 cm^−1^ were attributed to the stretching vibration of C-O-C group. The characteristic peak of absorption at 873.02 cm^−1^ was due to the variable-angle vibration of β-configuration C-H of pyrnoase, while the band at 819.07 cm^−1^ was attributed to the variable-angle vibration of C–H of furan ring. The absorption peak at 350–660 cm^−1^ indicated that it was a pyran-type polysaccharide. The ^1^H NMR spectrum of *RAMP* was shown in Fig. 2. In general, the vibrations at δ4.8-5.3 ppm indicate that the polysaccharide has an α-configuration, while the vibrations of a β-configuration are at δ4.0-4.8 ppm. The signals of *RAMP* showed that it was a polysaccharide with both α and β configurations, but the β-configuration was dominant.

### Stability and viscosity of *RAMP-O*

No separation was observed between aqueous and oil phases after *RAMP-O* was centrifuged for 15 min at 4000 rpm; it took 7.8 ± 0.4 s, 7.6 ± 0.3 s and 8.1 ± 0.4 s, respectively, for the oil emulsion containing 4,8 and 12 mg of *RAMP* per ml to flow out of the pipette, indicating that *RAMP-O* had a low viscosity.

### Irritation induced by subcutaneous injection of *RAMP-O* in the area of supramammary lymph node

**Fig. 3:**
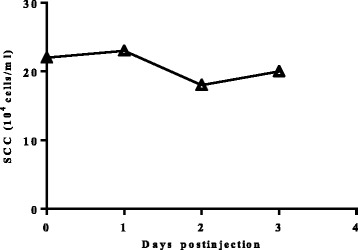
Irritancy of *RAMP-O* on milk SCC. SCC of composite milk in cows before and after subcutaneous injection of *RAMP-O* in left and right areas of supramammary lymph node in cow 435

Initially, we injected 32 mg of *RAMP-O* in a clinically healthy cow with milk SCC of 220,000/ml. No visible local reactions were observed at the injection site and no obvious change of SCC was detected in composite milk during 3 days following that injection (Fig. 3). Additionally, no side effects were found at the injection site throughout experiments 1 and 2.

### Simultaneous injections of *RAMP-O* in both left and right areas of the supramammary lymph node reduced SCC and NAGase activity in milk

In experiment 1, low (16 mg), middle (32 mg) and high (48 mg) doses of *RAMP-O* were used for injection in the area of the supramammary lymph node in groups 1 to 3. During three weeks after the treatment, both SCC and NAGase activity in milk were progressively declined in groups 1 to 3 (Tables 1 and 2). Although there was no significant difference among the three groups, the highest decrease was found in groups 2 and 3. Compared with the control, SCC decreased by 45.3 % and 46.0 %, respectively, in groups 2 and 3; NAGase decreased by 55.2 % and 60.3 %, respectively, in groups 2 and 3. Since there were no significant difference between the two groups, 32 mg of *RAMP-O* was used in our subsequent study.

**Table 1:** Influence of *RAMP-O* on composite milk SCC

RAMP-O (mg)	No. of cows	Weeks post treatment			
		0^†^	1	2	3
16	6	73.01 ± 10.05	65.79 ± 5.96	63.62 ± 17.94a*	54.65 ± 16.66a**
32	6	72.93 ± 18.42	62.31 ± 19.10	46.54 ± 25.14a	42.34 ± 21.30a*
48	6	71.48 ± 16.07	49.85 ± 23.49	45.01 ± 17.57A**	41.83 ± 14.81A*
Control	6	76.39 ± 14.38	77.20 ± 13.81	85.17 ± 12.15	77.47 ± 14.24

SCC of composite milk in cows before and after subcutaneous injection of *RAMP-O* in the areas of supramammary lymph node (mean ± S.E. 10^4^ cells/ml)

a*P* < 0.05, A*P* < 0.01, compared with the control in the same time

*P < 0.05, **P < 0.01, compared with pretreatment

^†^Pretreatment

**Table 2:** Influence of *RAMP-O* on composite milk NAGase

RAMP-O (mg)	No. of cows	Weeks post treatment			
		0^†^	1	2	3
16	6	52.07 ± 12.50	46.75 ± 10.09*	46.31 ± 9.79*	33.91 ± 9.27a*
32	6	52.46 ± 12.24	46.18 ± 9.49*	25.36 ± 6.77A**	23.79 ± 5.47A**
48	6	52.11 ± 11.35	27.27 ± 8.09A**	24.97 ± 6.85A**	21.09 ± 3.44A**
Control	6	52.66 ± 12.12	52.90 ± 12.07	55.09 ± 11.73	53.13 ± 11.81

NAGase of composite milk in cows before and after subcutaneous injection of *RAMP-O* in the areas of supramammary lymph node (mean ± S.E. U/L)

a*P* < 0.05, A*P* < 0.01, compared with the control in the same time

*P < 0.05, **P < 0.01, compared with pretreatment

^†^Pretreatment

Similar results were observed in experiment 2. Injection of *RAMP-O* (32 mg) significantly reduced SCC and NAGase in milk (Tables 3 and 4). In addition, significantly reduced SCC was also found in composite milk when compared to the control group (Table 5).

**Table 3:** Influence of *RAMP-O* on quarter milk SCC

Group	No. of quarters^††^	Weeks post treatment			
		0^†^	1	2	3
RAMP-O	14	63.39 ± 21.92	48.59 ± 27.52	40.18 ± 11.79a*	37.38 ± 11.36a*
Control	12	60.32 ± 10.20	54.53 ± 53.63	70.64 ± 67.24	64.37 ± 54.85

SCC of quarter milk in cows before and after subcutaneous injection of *RAMP-O* in the areas of supramammary lymph node (mean ± S.E. 10^4^ cells/ml)

a*P* < 0.05, compared with the control in the same time

*P < 0.05, compared with pretreatment

^†^Pretreatment

^††^The quarter had milk with SCC more than 500,000 cells/ml and positive bacterial examination before treatment

**Table 4:** Influence of *RAMP-O* on quarter milk NAGase

Group	No. of quarters^††^	Weeks post treatment			
		0^†^	1	2	3
RAMP-O	14	43.03 ± 26.69	23.75 ± 8.91a*	22.16 ± 9.59a**	17.86 ± 4.36a**
Control	12	38.01 ± 9.00	36.12 ± 28.89	76.30 ± 83.06**	61.59 ± 75.76

NAGase of quarter milk in cows before and after subcutaneous injection of *RAMP-O* in the areas of supramammary lymph node (mean ± S.E. U/L)

a*P* < 0.05, compared with the control in the same time

*P < 0.05, **P < 0.01, compared with pretreatment

^†^Pretreatment

^††^The quarter had milk with SCC more than 500,000 cells/ml and positive bacterial examination before treatment

**Table 5:** Influence of *RAMP-O* on SCC of composite milk

Group	No. of cows	Pretreatment	Posttreatment
RAMP-O	11	62.55 ± 25.38	29.21 ± 6.85a*
Control	11	56.66 ± 32.34	54.89 ± 11.88

SCC of composite milk in cows before and after subcutaneous injection of *RAMP-O* in the areas of supramammary lymph node (mean ± S.E. 10^4^ cells/ml)

a*P* < 0.05, compared with the control in the same time

*P < 0.05, compared with pretreatment

### Changes of bacteria infected quarters before and after treatment

Before treatment, bacteria were isolated from 21 quarter milk samples in *RAMP-O* treated cows while 23 quarters were found to have bacterial infection in the control (Table 6). There was no statistical difference between the 2 groups. The isolated bacteria were *Staphylococcus aureus*, *Streptococcus agalactiae*, *Streptococcus dysgalactiae*, *Streptococcus uberis*, coagulase-negative staphylococci (CNS), and others. After treatment, the quarters with bacterial infection were progressively reduced in *RAMP-O* treated cows and only 9 quarters were found to have bacterial infection, while no obvious change was found in the control group.

**Table 6:** Influence of *RAMP-O* on bacteria

Bacterium	Weeks post treatment							
	RAMP-O				Control			
	0^†^	1	2	3	0^†^	1	2	3
*Staphylococcus aureus*	7	6	4	3*	6	5	6	6
*Streptococcus agalactiae*	3	2	2	2	4	4	4	4
*Streptocossus dysgalactiade*	1	1	0	0	2	1	1	1
*Streptococcus uberis*	3	3	1	1	1	1	1	1
CNS^#^ and others	7	7	5	3*	10	9	10	10
Total	21	19	12*	9*	23	20	22	22

Milk samples with positive bacterial examination (tested quarters: 41 in *RAMP-O*; 44 in control)

*P < 0.05, compared with pretreatment

^†^Pretreatment

^#^CNS, coagulase negative staphylococci

## Discussion

In this study, we demonstrated a therapeutic effect of oil emulsified polysaccharide fraction of *Atractylodis macrocephalae* Koidz. (*RAMP-O*) on bovine subclinical mastitis. The use of immunomodulators to modulate bovine mammary gland has been previously reported. For example, it was found that intramammary administration of antibiotics in combination with recombinant bovine IL-2 for *Staphylococcus aureus* IMIs improved therapeutic efficacy by 20–30 % [23]. Ginseng saponins (GS) has been reported to stimulate lymphocyte proliferation and PMN phagocytosis from bovine peripheral blood and milk [13–15]. Recently, intramammary infusion of GS significantly increased IL-1α, IL-1β and TNF-α mRNA expression in cows at drying off [16, 17]. In the present study, the number of infected quarters was significantly reduced after *RAMP-O* treatment. As *RAMP-O* itself has no antibacterial activity, the diminished IMIs may be attributed to its immunomodulatory property.

*RAMP* was extracted from the rhizome of *Atractylodis macrocephalae Koidz.* The drug is traditionally orally administered for the treatment of diarrhea and infections in humans and animals. Zhou [24] reported a successful treatment of chronic respiratory infections using *Shen Ling Bai Zhu San* (codonopsis, atractylodes, poria, paeonia, nelumbo, dolichoris, coix, amomum, platycodon, citrus, aster, fritillaria and glycerrhiza) with an effective rate of 96 %. Oral administration of the soup or *RAMP* made from the rhizome has significantly enhanced the immune responses of mice to a model protein antigen ovalbumin or FMD vaccine [21, 25]. In 2013, Chai found an enhanced antibody response to ovalbumin by injection of *RAMP* with the antigen. Therefore, both oral and parental administrations of *RAMP* can improve the immune response. Chemical study has shown that *RAMP* is composed of rhamnose, arabinose, xylose, mannose, glucose, and galactose with molar ratios of 1.00: 2.49: 2.07: 4.94: 11.33: 1.35 [21]. In this study, we further analyzed the structure of *RAMP* using FTIR and ^1^H NMR spectra and the results indicated that it was a polysaccharide with both α- and β-configurations, while the main component was β-configuration. Previous investigations have shown that many polysaccharides isolated from herbs possess biological activities [26–28]. For example, the polysaccharides isolated from the root of *Astragalus membranaceus* have a backbone structure composed of glucoses and have been widely used for their immunomodulatory activities [25, 29, 30]. *RAMP* has been demonstrated to have the similar polysaccharide structure which may contribute to the therapeutic effect of *RAMP-O* on bovine subclinical mastitis.

Subcutaneous injection of *RAMP-O* in the area of supramammary lymph node significantly reduced milk SCC, NAGase and IMIs in subclinically infected lactating cows.

Mastitis is the most common disease of dairy cows. It is characterized by pathological alterations in the mammary tissues and compositional changes in the milk [31]. The economic losses caused by mastitis continue to bring heavy burden to dairy farm. Although current practices have reduced its occurrence, the disease remains heavily prevalent in many dairy herds [32]. Mastitis is mainly caused by invasion of bacteria in the mammary gland. When bacteria break through the physical barrier of teat canal, they encounter the second line of defense built by humoral and cellular protective factors of the mammary gland [12]. The humoral factors include immunoglobulin, complement, lactoferrin, lysozyme, lactoperoxidase system and so on while the cellular factors consist of macrophages, polymorphonuclear leukocytes (PMN), lymphocytes, etc. [33]. If the bacteria are virulent enough, they will settle in the mammary gland and establish the infection. The invading bacteria release chemotactic factors to attract large number of PMN flux into the mammary gland from blood stream, causing dramatically increased milk SCC up to millions of cells per ml of milk. Increased SCC is frequently used as an indicator for the intramammary inflammation [34]. In this study, we showed that subcutaneous injection of *RAMP-O* in the areas of supramammary lymph node in subclinically mastitic cows significantly reduced milk SCC in both experiments 1 and 2 with the largest scope in decrease found in the cows injected with 32 or 48 mg of *RAMP-O*. The decrease in milk SCC may be attributed to diminished IMIs as less infected quarters were observed in cows treated with *RAMP-O* than the control as demonstrated in experiment 2.

NAGase is an intracellular lysosomal enzyme which is released into milk from neutrophils during phagocytosis and cell lysis [35]. NAGase is mostly located in PMN and thus freezing and thawing of the milk samples are used for a maximal release of the enzyme [36]. The enhanced phagocytes in the mammary gland is correlated with the increase of the enzyme. Therefore, Milk NAGase is used as an indicator for IMIs. A successful antibiotic treatment could result in decrease of milk NAGase activity [37]. In the present study, significantly decreased NAGase (Tables 2 and 4) could be explained by reduced milk SCC which could be due to declined IMIs following *RAMP-O* treatment.

## Conclusions

After observing the above results, we concluded that subcutaneous injection of *RAMP-O* in the areas of supramammary lymph node had therapeutic value in the treatment of bovine subclinical mastitis by reducing SCC, NAGase and IMIs in milk. Considering the therapeutic effect and the cost of *RAMP-O*, 32 mg per dose was found most suitable to reduce milk SCC and NAGase. Therefore, *RAMP-O* needs further study for its use in treatment of bovine mastitis.

## Methods

### Extraction of polysaccharide fraction of *Atractylodis macrocephalae Koidz.* polysaccharides *(RAMP)*

Dried rhizome of *Atractylodis macrocephalae Koidz.* was purchased from Hu Qing Yu Tang Co. Ltd, Hangzhou, China. The polysaccharide fraction was extracted as per the method described earlier [21]. Briefly, the rhizome (100 g) was ground into powder and boiled twice under reflux for 2 h each time. The aqueous portion was filtered through filter paper. The filtrate was concentrated under reduced pressure, and then centrifuged at 3000 rpm for 15 min. Four volumes of 95 % ethanol were added to the supernatant, and kept overnight at 4 °C. The resulting precipitate was dissolved in distilled water, subjected to Macroporous Adsorption Resin column D101, and then washed with water. The collected elute was concentrated, dialyzed against distilled water (cut-off Mw 7000 Da) and lyophilized to afford a total polysaccharide (*RAMP*, light off-white powder, 8.53 g). Total sugar content was estimated by the phenol-sulfuric acid analysis using glucose as a standard [38]. Transmission Fourier transform infrared spectroscopy (FTIR) was conducted using Thermo NICOLET is5 (Nicolet Instrument, Thermo Company, USA). The *RAMP* samples were incorporated into KBr (spectroscopic grade) and pressed into a 1-mm pellet for FTIR measurement between 400 and 4000 cm^−1^. ^1^H NMR spectra of solutions in D_2_O was recorded at ambient temperature using Bruker AranceIII 400 M (Bruker, Switzerland).

### Rapeseed oil

Rapeseed oil was the product of the Shanghai Jiali Food Industry Co. Ltd, Shanghai, China, and manufactured according to Standard GB1536.

### Oil emulsified *Atractylodis macrocephalae Koidz.* polysaccharides (*RAMP-O*)

To produce oil emulsified *RAMP*, oil phase was prepared by mixing rapeseed oil with Span-80 to make an oil containing 14 % of Span-80; aqueous phase was prepared by addition of Tween-80 to *RAMP* solution to produce an *RAMP* solution containing 8 % of Tween-80 with 12, 24 or 36 mg of *RAMP* per ml. Then the oil phase was emulsified in the aqueous phase at 2: 1 (v/v) with a dispersing device (B. R. T TECHNOLOGY B25 Laboratory Series) to produce oil emulsified *RAMP* (*RAMP-O*). Each milliliter of *RAMP-O* contained *RAMP* of 4, 8 and 12 mg, respectively.

### Test of stability and viscosity

To test the stability of *RAMP-O*, sample was observed if there was any separation between aqueous and oil phases after centrifuged at 4000 rpm for 15 min; to test the viscosity of *RAMP-O*, the time required for 0.4 ml of *RAMP-O* sample to flow out of the 1-ml pipette positioned vertically was recorded.

### Irritancy test of *RAMP-O*

The cow 435 for the irritancy test was in her second mid lactation and clinically healthy. Each of four quarters had milk sample of SCC less than 500,000 cells/ml and negative bacteriological examination. Subcutaneous injection of *RAMP-O* at a dose of 32 mg in the area of supramammary lymph node was administered once after morning milking bilaterally. The injection sites were closely observed and milk samples were collected daily within 3 days after injection for SCC analysis.

### Selection of cows

This experiment was approved by the Institutional Animal Care and Use Committee at Zhejiang University and was conducted in accordance with the National Institutes of Health guidelines for the care and use of experimental animals. The therapeutic experiment was performed on a dairy farm in Jinhua, Zhejiang, China, having approximately 2,000 Holstein dairy cows that were machined-milked twice daily. The cows used in experiment 1 had SCC of composite milk more than 500,000 cells/ml, while the cows in experiment 2 had at least one quarter having positive bacterial examination and SCC more than 500,000 cells/ml.

### Experimental design

#### Experiment 1

A total of 24 lactating Holstein cows were used. They were randomly allocated into 4 groups with 6 animals in each. Groups 1 to 3 were subcutaneously injected with *RAMP-O* in the area of the supramammary lymph node after morning milking at the dose of 16, 32 or 48 mg, respectively. Each cow received a half dose injected at left side and the other half dose at right side. Group 4 received no treatment and served as a control. Composite milk was sampled from each cow before and 1, 2, 3 weeks after *RAMP-O* treatment for determination of SCC and NAGase.

#### Experiment 2

A total of 22 clinically healthy lactating Holstein cows with at least one quarter suffering from subclinical mastitis were used. The cows were randomly allocated into 2 groups: group 1 had 11 cows whose 14 of 41 quarters were subclinically infected; group 2 had 11 cows whose 12 of 44 quarters were subclinically infected. Groups 1 received a subcutaneous injection of *RAMP-O* (16 mg) in the left supramammary lymph node and the same amount of *RAMP-O* injected in the right supramammary lymph node after morning milking. The decision to use 32 mg of *RAMP-O* per dose was based on the balance between the cost of *RAMP-O* and the therapeutic effects found in experiment 1 in which no statistically significant difference was found between groups of 32 and 48 mg. Group 2 received no treatment and served as a control. Quarter milk samples were collected before and 1, 2, 3 weeks after *RAMP-O* treatment for bacteriological, SCC and NAGase analysis. Composite milk samples were collected before and one month after *RAMP-O* treatment.

### Estimation of somatic cell count (SCC)

For analysis of SCC, milk samples were heated to 40–42 °C. After shaking well, the samples were analyzed by Fossomatic Minor instrument (Foss Electric, Hillerod, Denmark).

### Bacteriological examination

Milk samples were streaked on blood agar plates and incubated aerobically at 37 °C for 24 to 48 h. After the culture, the plates were observed for primary isolation of bacteria. A milk sample was considered contaminated if 3 or more different bacterial colonies of bacteria were found. Afterward, a single colony on the blood agar was collected into nutrient broth medium and cultured for 18–24 h at 37 °C. Further identification of specific bacterial species such as staphylococci, streptococci, and gram-negative bacteria was carried out based on the methods described by the National Mastitis Council [39, 40].

### N-acetyl-β-D-glucosaminidase test

Milk samples were frozen and thawed for 3 times to liberate NAGase from cells and next centrifuged at 3,500 rpm for 20 min to eliminate cream layer. The skim milk was regulated to pH 4.6 by adding 10 % acetic acid and centrifuged at 3,500 rpm for 20 min to obtain whey. The NAGase in whey was measured by commercial kits (Nanjing Jiancheng Bioengineering Institute, Jiangsu, China) according to the manufacturer’s protocol. The OD value of paranitrophenol during the reaction (at 37 °C) between the 4-methylumbelliferyl-N-acetyl-β-glucosaminide substrate and the NAGase in the samples was estimated spectrophotometrically in triplicate at 400 nm. One unit of NAGase activity stands for the amount of paranitrophenol liberated from 1 liter of whey in 15 min at 37 °C.

### Statistical analyses

Data were analyzed by SPSS 20.0 software for windows and expressed as means ± standard error (S.E.). Independent-sample *t* test was performed to evaluate the differences between the data of *RAMP-O* treated group and the control. Paired-sample *t* test was carried out to compare the differences between the data of pretreatment and posttreatment. Chi-square analysis was used to compare the number of quarters infected with same bacteria in the same group between pretreatment and posttreatment. *P* values of less than 0.05 were considered statistically significant.

## Abbreviations

*RAMP*: *Atractylodis macrocephalae Koidz.* polysaccharides
*RAMP-O*: Oil emulsified *Atractylodis macrocephalae Koidz.* polysaccharides
SCC: Somatic cell count
IMIs: Intramammary infections
NAGase: N-Acetyl-β-D-glucosaminidase
CNS: Coagulase-negative staphylococci
GS: *Ginseng saponins*
FTIR: Transmission Fourier transform infrared spectroscopy
NMR: Nuclear magnetic resonance
PMN: Polymorphonuclear leukocyte
